# Protective effects of hydroalcoholic extract of *Rosa canina* L. fruit on cyclophosphamide-induced testicular toxicity in mice 

**DOI:** 10.22038/AJP.2022.20893

**Published:** 2023

**Authors:** Rahmatollah Parandin, Mahnaz Ghowsi, Ahmad Dadbod

**Affiliations:** 1 *Department of Biology, Payame Noor University, Tehran, Iran*; 2 *Department of Biology, Faculty of Sciences, Razi University, Kermanshah, Iran*; 3 *Department of Forensic Medicine, Judicature, Kermanshah, Iran*

**Keywords:** Rosa canina L., Cyclophosphamide, Oxidative stress, Nuclear factor erythroid-derived-2, Sperm, Testosterone

## Abstract

**Objective:**

Cyclophosphamide (CP)-induced testicular toxicity has been reported in recipient patients. The current study was designed to evaluate protective effects of hydroalcoholic extract of *Rosa canina* L. fruit (HARF) against CP-induced testicular toxicity in BALB/c mice.

**Materials and Methods:**

Thirty-five mice were divided into five groups as follows: group I (control), group II (CP, received CP 100 mg/kg on days 1, 8, 15, and 22), group III (CP + HARF 250 mg/kg), group IV (CP + HARF 500 mg/kg), and group V (CP + HARF 750 mg/kg). In the groups III, IV, and V that received CP, the HARF was simultaneously administered via intraperitoneal injections for 28 consecutive days starting from day 1. On the 29^th^ day, sperm parameters, stress oxidative biomarkers, and mRNA expression of *nuclear factor erythroid-derived-2 (Nrf2)* in testis tissue, as well as blood testosterone were evaluated.

**Results:**

The CP exposure decreased sperm parameters, superoxide dismutase (SOD) activity, testosterone, and *Nrf2* mRNA expression levels and increased the malondialdehyde (MDA). HARF at the dose of 500 mg/kg improved sperm count and viability and increased SOD and catalase activities, glutathione peroxidase (GPx) activity, testosterone level, and *Nrf2* expression and reduced MDA. Also, HARF at the dose of 750 mg/kg improved sperm parameters and increased SOD, catalase, and GPx activities, total testosterone level, and *Nrf2* expression, and reduced MDA in comparison with the CP group.

**Conclusion:**

According to our findings, HARF at the doses of 500 and 750 mg/kg inhibited the ruinous effects of CP on the reproductive system in mice.

## Introduction

Cyclophosphamide (CP) (*N*, *N*-bis (2-chloroethyl)-2-oxo-1,3,2λ^5^-oxazaphosphinan-2-amine) is a chemotherapeutic agent that is applied as an anticancer and immunosuppressive agent for treatment of different diseases. This alkylating agent has toxic side-effects in various organs of recipient patients including the testes and may cause male infertility (Fusco et al., 2021 ▶; Hosseini et al., 2018 ▶). CP causes infertility by inducing oxidative stress via inducing an increase in the malondialdehyde (MDA) levels and reducing superoxide dismutase (SOD) and catalase (CAT) activity and glutathione peroxidase (GPx) levels in the testes. Also, it impairs genes expression during different stages of development of spermatocytes (Ghobadi et al., 2017 ▶; Novin et al., 2020 ▶; Sakr et al., 2012 ▶). CP may result in infertility due to extreme oxidative stress and apoptosis in the testes induced by its metabolite, acrolein. Acrolein can generate toxic reactive oxygen species (ROS) that inhibit different enzymes, damage membrane and DNA, and induce lipid peroxidation which cause infertility (Fusco et al., 2021 ▶). Alteration in the membrane potential induced by oxidative stress-induced lipid peroxidation (LPO) can decrease the energy production capacity and increase the electron leakage and ROS production. This situation can lead to damage to the DNA, RNA transcripts, and telomeres, disturbance of membrane fluidity and structure of sperm, reduction of sperm motility, and caspase activation, and apoptosis (Alahmar, 2019 ▶; Bisht et al., 2017 ▶; Nowicka-Bauer and Nixon, 2020 ▶). The primary enzymes including SOD, glutathione (GSH), and CAT as well as inducible phase II detoxifying enzymes including heme oxygenase-1 (HO-1) and NAD(P)H dehydrogenase (quinone) 1 (NQO1) through activation of nuclear transcription factor-erythroid 2 related factor (Nrf2) form a network against oxidative stress (Fusco et al., 2021 ▶). The Nrf2 pathway is an important regulator of cellular defense during oxidative stress (Darbandi et al., 2019 ▶). In the animal cells, Nrf2 is a central regulator of basal and inducible antioxidant mechanisms (Shanmugam et al., 2017 ▶). Although, under physiological conditions, Nrf2 is placed in the cytoplasm and it is connected to Kelch-like ECH-associating protein 1 (Keap1) that is a negative regulator for Nrf2, upon oxidative stress conditions, Nrf2 is dissociated from the Keap1–Nrf2 complex and as a result, it can enter the nucleus. There, it consecutively binds to the antioxidant response element (ARE) that is a regulatory enhancer region within gene promoters (Fusco et al., 2021 ▶). Therefore, Nrf2 can induce the expression of several detoxifying and antioxidant enzyme genes, which defend cells against oxidative stress (Fusco et al., 2021 ▶; Kim and Jang, 2014 ▶; Ku et al., 2006 ▶)

Recently, use of natural antioxidants, especially medicinal products with plant origin for reduction of free radicals production and oxidative stress in testicular tissue and thereby, improving fertility has attracted great attention worldwide (Almeer et al., 2018 ▶). Induction of antioxidant system, reduction of LPO, and improvement of the damaged testis are among the beneficial properties of these natural products (Attia et al., 2012 ▶). *Rosa canina* Linnaeus (a member of the Rosaceae family known as dog rose hips) is used as a traditional medicine for medicinal purposes and some physiological disorders (Sadeghi et al., 2021 ▶). Various extracts of this plant have numerous phytochemical components such as polyphenols, flavonoids, tannins, carotenoids, fatty acids, vitamins particularly vitamin C, B1, B2, K, and E, minerals and show high antioxidant properties (Bussmann et al., 2011 ▶; Roman et al., 2013 ▶) that are protective against oxidative stress and systemic inflammation (Fusco et al., 2021 ▶). The main components of the rose hip extract are quercitrin, hyperoside, vitamin C quercetin-3-o-glucoside, beta-sitosterol, folic acid, and beta-carotene (Nađpal et al., 2016 ▶). Particularly, the fruit (hips) of *R. canina* L. is rich in vitamin C with strong antioxidant power (Winther et al., 2016 ▶). 

Considering widespread application of anticancer and immunosuppressive agent CP for treatment of various malignant and non-malignant tumors and its side effects on male reproductive organs (Fusco et al., 2021 ▶) and due to the medicinal properties of *R. canina* L. fruit, the focus of the current study was to evaluate whether hydroalcoholic extract of *R. canina* L. fruit (HARF) can restrict the CP-induced oxidative stress in the testicular tissue. Also, the mRNA expression of *Nrf2* in the testes was evaluated.

## Materials and Methods

**Materials and the extract**

All compounds were prepared from Sigma-Aldrich except otherwise said. To prepare HARF, fresh fruits of *R. canina* L. were collected from Kermanshah, Iran in summer 2020. The collected fruits were identified and approved by an expert at Payame Noor University, Kermanshah, Iran (voucher specimen 1397.2). The fruits were shade dried and crushed into a fine powder by a grinder. Then, 60 g from fruit powder was mixed with 150 ml of methanol and 150 ml of water in a Soxhlet apparatus for 10 hr. Then, the suspension was refined and concentrated by a rotary evaporator at 40°C. About 19 g of the extracted dry matter was reconstructed to prepare a solution of 65 mg/ml in distilled water just before the onset of the experiments (Sadeghi et al., 2016 ▶; Tayefi‐Nasrabadi et al., 2012 ▶). The extract was stored at −20°C. Three doses 250, 500, and 750 mg/kg of HARF were selected (Gholampour et al., 2012 ▶; Sadeghi et al., 2016 ▶; Tayefi‐Nasrabadi et al., 2012 ▶) based on the previous reports (Sadeghi et al., 2021 ▶) these doses from HARF had protective effects against vancomycin-induced nephrotoxicity.

**The experimental design**

Thirty-five healthy male laboratory mice (BALB/c strain) weighing about 26-31 g (10-11 months of age) were kept under controlled conditions (12 hour light/dark cycle, 21±2°C, and relative humidity 50±5%) with free access to rodent pellet and tap water. All animals were acclimatized for 14 days in the animal house before treatments. All the protocols applied in the present study were approved by the ethical guidelines for animal studies advised by the Research Ethics Committee of Razi University, Kermanshah, Iran (No. IR.RAZI.REC.1399.042). 

The animals were randomly divided to 5 groups (n=7) as follows: I) Control group: animals were subjected to injections of normal saline for 4 weeks; II) CP: The animals received only CP 100 mg/kg, i. p. once daily on days 1, 8, 15, and 22 (Salimnejad et al., 2018 ▶); and groups III), IV) and V) CP+HARF 250, 500, and 750: animals were subjected to CP injections as described for group II and treated by i. p. injection of HARF (dissolved in saline) at doses of 250, 500, and 750 mg/kg, respectively 1 hour after CP-injections for 28 consecutive days starting from day 1, once a day (Gholampour et al., 2012 ▶; Tayefi‐Nasrabadi et al., 2012 ▶). 

**Collection of tissues samples**

Twenty-four hours after the last injection, fasted mice were anaesthetized by using a combination of ketamine (50 mg/kg) (Alfasan, Netherlands) and xylazine (7 mg/kg) (Alfasan, Netherlands). The blood samples were prepared via cardiac puncture. The right epididymis was cut for analyzing sperm parameters and the testes were frozen and kept at -80°C to evaluate *Nrf2* mRNA expression and antioxidant activity. 

**Evaluation of sperm**

The right caudal epididymis was sliced and applied for sperm analysis. For this purpose, the tissues were teased and homogenized in 5 mL of human tubal fluid medium (HTF). The resulting mixture was incubated in A CO2 incubator at 37˚C for 30 min to release sperms from the tissue. To assess the sperm motility, 10 µl of the sperm diluted stock was put on a Neubauer slide. A minimum of 5 microscopic fields and at least 200 sperms were assessed for each sample. Sperm motility (% of sperm that display motility of any form) was calculated by microscopic observation (400× magnification)(Luo et al., 2015 ▶). To evaluate the sperm viability, 20 µl of sperm suspension was added to the same amount from 0.05% eosin‐nigrosin stain. After 2 min of incubation at 25°C, slides were observed under a light microscopic (400× magnification). The dead sperms were pink but the live ones had no color (Luo et al., 2015 ▶). In each sample, at least 200 sperms were evaluated and the viability percentage was calculated. In order to count the sperms, 10 µl of sperm suspension was added to 190 µl of distilled water and 10 µl of this diluted solution was dropped on a Neubauer slide. The chambers were evaluated by microscopic observation (400× magnification). The counts of sperms are expressed as sperm/mL (Almeer et al., 2018 ▶; Wyrobek et al., 1983 ▶). 

**Assessment of antioxidant activity in testis tissue**

The testis samples were washed with distilled water and homogenized in ice-cold 50 mM sodium phosphate buffer (pH 7.4) and 0.1 mM EDTA; then the samples were centrifuged (1000 *g* for 20 min), and supernatant fluids were used as stock and kept at -18°C for assessment of antioxidant enzymes and lipid peroxidation levels (Attia et al., 2012 ▶). 

**Malondialdehyde (MDA) levels**

Under oxidative stress conditions, unsaturated membrane lipids are influenced by progressive deterioration by lipid peroxidation (Fusco et al., 2021 ▶). The testicular LPO parameter was estimated by determination of MDA levels. Briefly, 250 µl from the stock supernatant was added to 1 ml thiobarbituric acid 0.6% and 1.5 ml phosphoric acid 1% (pH 2). The solution was heated in a boiling water bath for 25 min. The samples were cooled, mixed with 2.5 ml of butanol, and centrifuged for 10 min (4000 rpm). Then, the absorbance was determined at 532 nm (Young and Trimble, 1991 ▶).

**Superoxide dismutase (SOD) activity**

To measure the specific activity of testicular SOD, 10 µl of stock supernatant was added to 970 µl EDTA-sodium carbonate buffer 0.05 M (pH 10.2). Then, 20 µl epinephrine 30 mM was added to the solution and SOD activity was determined at 480 nm (4 min) (Liu et al., 1977 ▶). 

**Glutathione peroxidase (GPx) activity**

In this step, 200 µl from stock supernatant was added to a mixture of 1 ml of phosphate buffer (75 mM, pH 7), 10 ml glutathione 150 mM, 10 ml glutathione reductase 340 U/ml, 30 ml EDTA 25 mM, 30 ml NADPH 5 mM, 10 ml Triton X-100 20%, and 50 µl of H_2_O_2_7.5 mM. GPx activity was determined at 340 nm (3 min) (Almeer et al., 2018 ▶). 

**Catalase (CAT) activity**

To determine CAT activity, 25 µl of stock supernatant and 625 µl from potassium phosphate buffer 50 mM (pH 5.0) were mixed with 100 µl H_2_O_2_ 5.9 mM. The activity of testicular CAT was evaluated spectrophotometrically at 240 nm (Shah and Khan, 2017 ▶). 

**Blood testosterone assay**

For testosterone estimation, the serum samples were separated from blood by 15 min of centrifugation at 3000 *g*, and kept at −20°C. Concentrations of total testosterone in serum samples were measured by RIA using ^125^I-testosterone Radioimmunoassay Kits (Beijing North Institute of Biological Technology, Beijing, China) according to the manufacturer’s manual. The intra- assay coefficients of variation were 6.9% and inter-assay variation was 7.2%.

**Isolation of RNA and real-time qPCR analysis**

Total RNA from testis tissue was extracted using a TRIzol reagent kit (Invitrogen, USA) and quantified in an ND-100 spectrophotometer. RNA samples were reverse transcribed using the cDNA synthesis Kit (Fermentas, California, U.S.A.) according to the manufacturer’s guidance. In order to eliminate genomic DNA, RNA was treated with DNaseІ (RNase-free) (TaKaRa, Dalian, China). The SYBR Green PCR master mix (Fermentas, California, U.S.A.) was used for real-time quantitative polymerase chain reaction (qPCR) analysis and the assays were performed. The housekeeping gene was *GAPDH* and the target gene was *Nrf2*. The PCR reactions were done in triplicates and 2 μl cDNA, 3 μl water, and 6 μl 2 X SYBR Green Master mixes (SYBR premix Ex Taq TMII (Takara Holdings Inc., Kyoto, Japan) and primer pairs at 5 pmol concentrations in a final volume of 12 μl were mixed. The amplification condition was as follows: 95°C for 30 sec, denaturation at 95 °C for 5 sec, annealing at 58°C for 30 sec, and extension at 72°C for 30 sec with 42 repeated amplification cycles. The green fluorescence at the end of each extension step was measured. The sequences of the primers were as follows: *GAPDH* (Forward: ′CCTTCATTGACCTCAACTAC; Reverse: 5′ATGACAAGCTTCCCATTCTC) and *Nrf2 *(Forward:5′CATCGTGGGCCGCTCTA3′; Reverse:5′TTCTGGGCGGCGACTTTATT3′). Relative expression was quantified by using threshold cycle (Ct) method, and expression fold-change was calculated by normalization to the Ct of housekeeping gene *GAPDH* according to the 2^-ΔΔCt method (Schmittgen and Livak, 2008 ▶).

**Statistical analysis**

The statistical analysis performed by using SPSS (version 16) and one-way ANOVA followed by Tukey's *post hoc* test. Data are expressed as mean±SEM and a p<0.05 indicates statistically significant difference between groups.

## Results

**Impact of HARF administration on sperm parameters and total testosterone**

CP administration altered the sperm parameters considerably. The sperm motility in the CP (p<0.001), CP+HARF 250 (p<0.001), and CP+HARF 500 groups (p<0.05) were decreased compared to the controls but, in the CP+HARF 750 group, this parameter was similar to the control group (p<0.05). The sperm motility level in the CP+HARF 750 group was higher than that of the CP group (p<0.05). 

The sperm viability in the CP (p<0.001), and CP+HARF 250 (p<0.001) and 500 groups (p<0.01) was decreased in comparison with the control group (p<0.05). The sperm viability in the CP+HARF 500 and 750 groups was significantly higher than the CP group (p<0.001). The sperm viability in the CP+HARF 750 group was similar to the control group (p<0.05).

**Figure 1:**
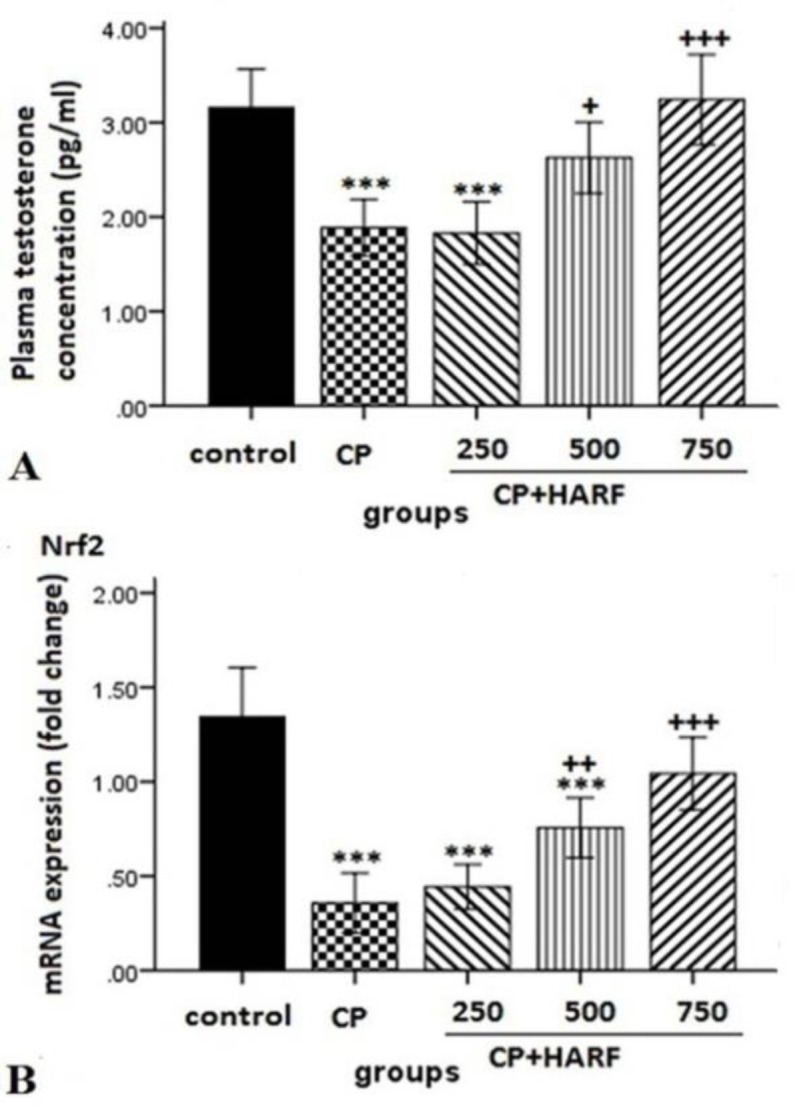
Effect of hydroalcoholic extract of *R. canina* L. fruit (HRF) and cyclophosphamide (CP) on the plasma total testosterone concentration (A) and mRNA expression of *Nrf2 *in the testis of mice (B). Data represent the mean±SEM (n=7). ***p<0.001 *vs.* the control group; +p<0.05 *vs.* the CP group; ++p<0.01 *vs.* the CP group +++p<0.001 vs. the CP group. One-way ANOVA. Group CP, received CP (100 mg/kg) on days 1, 8, 15, and 22; In the groups CP + HARF 250, CP + HARF 500 and CP + HARF 750, CP was injected to the animals on days 1, 8, 15, and 22 and the HARF (dissolved in saline) at doses of 250, 500, and 750 mg/kg was administered via intraperitoneal injections for 28 consecutive days starting from day 1

The sperm count in the CP and CP+HARF 250 groups was lower than that of the control group (p<0.001). This parameter in the CP+HARF 500 and 750 groups was significantly higher than that of the CP group (p<0.001) (Table 1). The total testosterone concentrations of CP and CP+HARF 250 groups were lower than the control group (p<0.001). The total testosterone levels in the CP+HARF 500 group (p<0.05) and CP+HARF 750 group (p<0.001) were increased in comparison with the CP group. The total testosterone levels in the CP+HARF 500 group (p<0.05) and CP+HARF 750 group (p<0.001) were higher than that of the CP+HARF 250 groups (Figure 1A). 

**MDA levels**

The animals of the CP (p<0.001), CP+HARF 250 (p<0.001) or CP+HARF 500 (p<0.01) groups had a significant increase in the amount of MDA in the testes tissue when they were compared to the animals of control group. Also, the levels of MDA in the CP+HARF 750 (p<0.01) were lower than the levels of MDA in the CP group (p<0.001). 

The levels of MDA in the CP+HARF 750 group were similar to the control group (p<0.05), suggesting that HARF 750 administration was capable of restoring the levels of MDA to those of the control group (Table 1). 

**SOD** **activity**

The SOD activity of the CP (p<0.001) and CP+HARF 250 groups (p<0.01) was lower than that of the control group (p<0.01). But this parameter in the CP+HARF 750 group was higher than that of the control group (p<0.05). The results showed that the SOD activity in the CP+HARF 500 (p<0.01) and CP+HARF 750 (p<0.001) groups was significantly increased when compared to the CP group (Table 1).

**CAT activity**

CAT activity in the CP and CP+HARF 250 groups was not significantly different from that of the control group. In the CP+HARF 750 group, a significant increase in the CAT activity was seen in comparison with the control group (p<0.05). In the CP+HARF 500 (p<0.01) and CP+HARF 750 (p<0.001) groups, CAT activity was increased in comparison to the CP group (Table 1).

**GPx activity**

A decrease in GPx activity was observed in the CP group (p<0.05) in comparison with the control group. In the CP+HARF 250 group, the GPx activity was not significantly different from that of the control group. Also, the results showed that in the CP+HARF 750 group, the GPx activity was significantly increased (p<0.01) in comparison with the control group. In the CP+HARF 500 (p<0.001) and CP+HARF 750 (p<0.001) groups, a significant increase in the GPx activity was observed compared to the CP group (Table 1).

***Nrf2*** **mRNA expression**

The mRNA expression levels of *Nrf2* in the CP, CP+HARF 250, and CP+HARF 500 groups were significantly lower than that of the control group (p<0.001). In the CP+HARF 500, and CP+HARF 750 groups, the *Nrf2* mRNA expression was increased in comparison with the CP group ((p<0.01) and (p<0.001), respectively) (Figure 1B).

**Table 1:** Impact of *Rosa canina* L. fruit extract on oxidative stress parameters in the testes

Groups(n=7)	Control	CP	CP+HARF 250	CP+HARF 500	CP+HARF 750
Sperm motility (%)	86.43±4.32	50.71±4.93***	51.43±4.84***	68.57±3.03*	78.58±2.83^+++^
Sperm viability (%)	91.43±2.10	52.71±4.36***	57.14±3.06***	72.14±2.64**^,+++^	82.14±3.25^+++^
Sperm count (× 10^6^)	21.57±0.99	11.71±0.61***	13.43±0.68***	18.71±1.02^+++^	21.67±1.14^+++^
MDA (nmol/mg protein)	2.73±0.13	5.31±0.18***	5.48±0.19***	3.67±0.17**^,+++^	3.17±0.11^+++^
SOD activity (U/mg protein)	26.71±1.54	19.28±1.27**	19.28±1.11**	26.71±0.84^++^	32.86±1.45*^,+++^
Catalase activity (U/mg protein)	19.14±1.50	13.57±1.07	17.28±1.06	22.43±1.76^++^	26.28±2.05*^,+++^
GPx activity (U/mg protein)	17.14±0.86	12.43±0.84*	13.86±0.99	21.43±1.54^+++^	23.86±1.20**^,+++^

CP, cyclophosphamide; HARF, hydroalcoholic extract of *R. canina* L. fruit. *p<0.05, **p<0.01, and ***p<0.001 vs. the control group, respectively; +++p<0.001 and ++p<0.01 vs. the CP group. One-way ANOVA. Group CP, received CP (100 mg/kg) on days 1, 8, 15, and 22; In the groups CP + HARF 250, CP + HARF 500 and CP + HARF 750, CP was injected to the animals on days 1, 8, 15, and 22 and the HARF (dissolved in saline) at doses of 250, 500, and 750 mg/kg was administered via intraperitoneal injections for 28 consecutive days starting from day 1.

## Discussion

The chemotherapeutic CP is widely administered to treat several types of cancers and autoimmune diseases (Ghobadi et al., 2017 ▶). Here, we investigated the effects of the hydroalcoholic extract prepared from of *R. canina* L. fruit (HARF) on testicular toxicity and oxidative stress induced by CP in mice. CP is rapidly metabolized to aldophosphamide mustard and acrolein, which induce oxidative stress and damage DNA, lipids, proteins, and DNA in the cells (Fusco et al., 2021 ▶; Korkmaz et al., 2007 ▶). Here, the administration of CP decreased the sperm motility, viability, and count. Previous studies reported that CP-induced toxicity in the testis is due to the increase in the ROS and free radical levels in this tissue and treatment with CP significantly increased MDA levels and declined SOD and CAT activity as well as GPx levels (Novin et al., 2020 ▶; Sakr et al., 2012 ▶).Our findings also revealed a remarkable raise in the MDA, as well as a reduction in SOD, GPx, and CAT activity in the testes of the CP group. Our results show that oxidative stress induced by CP affects the sperm parameters. Similarly, Ghobadi et al. (2017) ▶ reviewed that CP induces its effects mostly via an increase in the oxidative stress and alteration of the gene expression in sperms and these effects could be suppressed by the treatments with antioxidant agents (Ghobadi et al., 2017 ▶). Although many studies have suggested that CP disrupts the balance between free radicals and antioxidants (Novin et al., 2020 ▶; Sakr et al., 2012 ▶), but the exact mechanism of CP’s reproductive toxicity needs more elucidation. An increase in intracellular oxidative burden may result in the lipid peroxidation and loss of membrane integrity that is associated with increased permeability, reduced sperm motility, structural damage of DNA, and apoptosis (Alahmar, 2019 ▶). Due to the presence of abundant polyunsaturated fatty acids in plasma membrane of sperms, their function and testis cell physiology is very sensitive to high levels of ROS and thereby, oxidative stress condition can impair spermatogenesis and sperm quality and affect the sperm viability/motility (Aprioku, 2013 ▶). 

Also, in the present study, similar to study of Elangovan et al. (Elangovan et al., 2006 ▶), CP injection decreased the testosterone levels. Our findings showed that administration of HARF at doses 500 and 750 mg/kg considerably reversed the CP-induced testicular toxicity and increased the antioxidant capacity of testis tissue and total testosterone levels. Our study clearly showed that treatment with HARF significantly increased the sperm motility, viability, and count, as well as the total testosterone concentration in the mice with CP-induced testicular toxicity. Previous studies have shown that use of antioxidant supplementation can improve some seminal fluid parameters (Alahmar, 2018 ▶; Hamzeh et al., 2019 ▶; Imamovic Kumalic and Pinter, 2014 ▶; Sakr et al., 2012 ▶). Also, the aqueous extract of *R. canina* L. has shown protective properties against doxorubicin-induced testicular toxicity (Nowrouzi et al., 2019 ▶). 

The Nrf2 regulates oxidative and apoptotic responses in different cells and diseases (Fusco et al., 2021 ▶; Kim et al., 2020 ▶). In the present study, the levels of *Nrf2* mRNA expression in the testes were found to be decreased in the CP group. These findings are in accordance with findings of previous studies that showed that CP therapy declined the levels of Nrf2 and interrupted the Nrf2 downstream pathway molecules such as HO-1 and SOD and the physiological antioxidant response (Fusco et al., 2021 ▶; Maremanda et al., 2014 ▶). Here, the result of analysis of *Nrf2* gene expression showed that treatment of animals with HARF 500 and 750 mg/kg considerably increased *Nrf2* mRNA expression in the mice treated with CP. This finding is in line with previous studies (Almeer et al., 2018 ▶; Elmallah et al., 2017 ▶). Thus, a decrease in the CP-induced oxidative stress by administration of an antioxidant agent such as HARF can improve the testicular dysfunction. Similarly, one study has reported that methanolic extract of *Fragaria ananassa* Duch. (Rosaceae family) improved cadmium-induced oxidative damage in rat testes (Elmallah et al., 2017 ▶). The antioxidant compounds of HARF such as vitamins (C, E, thiamine, riboflavine, and niacin), polyphenols, flavonoids, carotenoids and minerals could play an important role in reduction of oxidative stress and protection against CP-induced testicular toxicity. Our results showed that treatment with HARF decreased the MDA levels and it significantly increased the activity of SOD, GPx, and CAT. In line with our findings, in a previous study by Naziroğlu (2003) ▶ on streptozotocin-induced diabetic rats, it has been demonstrated that treatment with vitamins C and E decreased the testicular LPO (Naziroğlu, 2003 ▶). 

*Rosa canina* fruits contain phenolic acids, proanthocyanidins, tannins, flavonoids, fatty acids, pectines, carotenoids, and fruit acids (ascorbic acid, malic acid, and citric acid) (Roman et al., 2013 ▶). 

On the other hand, as mentioned above, the deleterious effects of CP on the testis tissue is via induction of oxidative stress. Our findings showed that administration of HARF with antioxidant properties may help attenuation of the deleterious impact of CP on the testes and sperm parameters and may improve oxidative endogenous defense and as a consequence, it may be helpful in reduction of toxic side effects of CP on the reproductive system. However, in the present study, we did not perform histopathological examination of testes sections to confirm our findings and more research is needed. Also, one limitation of this study was the lack of characterization of the exact for its main ingredients. Our findings emphasize the need for the development of new therapeutic interventions to prevent the burden of toxic side effects CP in the male reproductive system. Because of effects of sperm membrane integrity and the functions of sperm metabolic enzymes such as phosphofructokinase (PFKP); aldolase A, fructose-bisphosphate (ALDOA), phosphoglycerate kinase (PGK), etc. on the sperm motility (Nowicka-Bauer and Nixon, 2020), it is suggested that sperm membrane integrity function of these enzymes be examined in the future studies. 

## Conflicts of interest

The authors have declared that there is no conflict of interest.

## References

[B1] Alahmar AT (2018). The effects of oral antioxidants on the semen of men with idiopathic oligoasthenoteratozoospermia. Clin Exp Reprod Med.

[B2] Almeer RS, Soliman D, Kassab RB, AlBasher GI, Alarifi S, Alkahtani S, Ali D, Metwally D, Abdel Moneim AE (2018). Royal jelly abrogates cadmium-induced oxidative challenge in mouse testes: involvement of the Nrf2 pathway. Int J Mol Sci.

[B3] Aprioku JS (2013). Pharmacology of free radicals and the impact of reactive oxygen species on the testis. J Reprod Infertil.

[B4] Attia AA, ElMazoudy RH, El-Shenawy NS (2012). Antioxidant role of propolis extract against oxidative damage of testicular tissue induced by insecticide chlorpyrifos in rats. Pestic Biochem Physiol.

[B5] Bisht S, Faiq M, Tolahunase M, Dada R (2017). Oxidative stress and male infertility. Nat Rev Urol.

[B6] Bussmann RW, Swartzinsky P, Worede A, Evangelista P (2011). Plant use in odo-bulu and demaro, Bale region, Ethiopia. J Ethnobiol Ethnomed.

[B7] Darbandi M, Darbandi S, Agarwal A, Baskaran S, Sengupta P, Dutta S, Mokarram P, Saliminejad K, Sadeghi MR (2019). Oxidative stress‐induced alterations in seminal plasma antioxidants: Is there any association with keap1 gene methylation in human spermatozoa?. Andrologia.

[B8] Elangovan N, Chiou T-J, Tzeng W-F, Chu S-T (2006). Cyclophosphamide treatment causes impairment of sperm and its fertilizing ability in mice. Toxicology.

[B9] Elmallah MI, Elkhadragy MF, Al-Olayan EM, Abdel Moneim AE (2017). Protective effect of Fragaria ananassa crude extract on cadmium-induced lipid peroxidation, antioxidant enzymes suppression, and apoptosis in rat testes. Int J Mol Sci.

[B10] Fusco R, Salinaro AT, Siracusa R, D’Amico R, Impellizzeri D, Scuto M, Ontario ML, Crea R, Cordaro M, Cuzzocrea S (2021). Hidrox® counteracts cyclophosphamide-induced male infertility through NRF2 pathways in a mouse model. Antioxidants.

[B11] Ghobadi E, Moloudizargari M, Asghari MH, Abdollahi M (2017). The mechanisms of cyclophosphamide-induced testicular toxicity and the protective agents. Expert Opin Drug Metab Toxicol.

[B12] Gholampour F, Javadifar TS, Karimi S, Eslam-Zadeh T, Owji SM (2012). The effects of the hydroalcohol extract of Rosa canina L fruit on ischemic acute renal failure in Wistar rats. Comp Clin Path.

[B13] Hamzeh M, Hosseinimehr SJ, Karimpour A, Mohammadi HR, Khalatbary AR, Amiri FT (2019). Cerium oxide nanoparticles protect cyclophosphamide-induced testicular toxicity in mice. Int J Prev Med.

[B14] Hosseini A, Zare S, Borzouei Z, Pakdel FG (2018). Cyclophosphamide-induced testicular toxicity ameliorate by American ginseng treatment: An experimental study. Int J Reprod Med.

[B15] Imamovic Kumalic S, Pinter B (2014). Review of clinical trials on effects of oral antioxidants on basic semen and other parameters in idiopathic oligoasthenoteratozoospermia. Biomed Res Int.

[B16] Kim J-K, Jang H-D (2014). Nrf2-mediated HO-1 induction coupled with the ERK signaling pathway contributes to indirect antioxidant capacity of caffeic acid phenethyl ester in HepG2 cells. Int J Mol Sci.

[B17] Kim S, Viswanath ANI, Park J-H, Lee HE, Park AY, Choi JW, Kim HJ, Londhe AM, Jang BK, Lee J (2020). Nrf2 activator via interference of Nrf2-Keap1 interaction has antioxidant and anti-inflammatory properties in Parkinson's disease animal model. Neuropharmacology.

[B18] Korkmaz A, Topal T, Oter S (2007). Pathophysiological aspects of cyclophosphamide and ifosfamide induced hemorrhagic cystitis; implication of reactive oxygen and nitrogen species as well as PARP activation. Cell Biol Toxicol.

[B19] Ku BM, Joo Y, Mun J, Roh GS, Kang SS, Cho GJ, Choi WS, Kim HJ (2006). Heme oxygenase protects hippocampal neurons from ethanol-induced neurotoxicity. Neurosci Lett.

[B20] Liu J, Simon LM, Phillips JR, Robin ED (1977). Superoxide dismutase (SOD) activity in hypoxic mammalian systems. J Appl Physiol.

[B21] Luo T, Zou Q-x, He Y-q, Wang H-f, Li N, Zeng X-h (2015). Matrine inhibits mouse sperm function by reducing sperm [Ca2+] i and phospho-ERK1/2. Cell Physiol Biochem.

[B22] Maremanda KP, Khan S, Jena G (2014). Zinc protects cyclophosphamide-induced testicular damage in rat: Involvement of metallothionein, tesmin and Nrf2. Biochem Biophys Res Commun.

[B23] Nađpal JD, Lesjak MM, Šibul FS, Anačkov GT, Četojević-Simin DD, Mimica-Dukić NM, Beara IN (2016). Comparative study of biological activities and phytochemical composition of two rose hips and their preserves: Rosa canina L and Rosa arvensis Huds. Food chem.

[B24] Naziroğlu M (2003). Enhanced testicular antioxidant capacity in streptozotocin-induced diabetic rats. Biol Trace Elem Res.

[B25] Novin MG, Golmohammadi MG, Sagha M, Ziai SA, Abdollahifar MA, Nazarian H (2020). Protective effect of gallic acid on testicular tissue, sperm parameters, and dna fragmentation against toxicity induced by cyclophosphamide in adult NMRI mice. Urol J.

[B26] Nowicka-Bauer K, Nixon B (2020). Molecular changes induced by oxidative stress that impair human sperm motility. Antioxidants.

[B27] Nowrouzi F, Azadbakht M, Kalehoei E, Modarresi M (2019). Protective effect of Rosa Canina extract against doxorubicin-induced testicular toxicity in mice. Braz Arch Biol Technol.

[B28] Roman I, Stănilă A, Stănilă S (2013). Bioactive compounds and antioxidant activity of Rosa canina L biotypes from spontaneous flora of Transylvania. Chem Cent J.

[B29] Sadeghi H, Hosseinzadeh S, Touri MA, Ghavamzadeh M, Barmak MJ (2016). Hepatoprotective effect of Rosa canina fruit extract against carbon tetrachloride induced hepatotoxicity in rat. Avicenna J Phytomed.

[B30] Sadeghi H, Karimizadeh E, Sadeghi H, Mansourian M, Abbaszadeh-Goudarzi K, Shokripour M, Asfaram A, Doustimotlagh AH (2021). Protective effects of hydroalcoholic extract of Rosa canina fruit on vancomycin-induced nephrotoxicity in rats. J Toxicol.

[B31] Sakr SA, Mahran HA, Abo-El-Yazid SM (2012). Effect of fenugreek seeds extract on cyclophosphamide-induced histomorphometrical, ultrastructural and biochemical changes in testes of albino mice. Toxicol Ind Health.

[B32] Salimnejad R, Rad JS, Nejad DM (2018). Protective effect of ghrelin on oxidative stress and tissue damages of mice testes followed by chemotherapy with cyclophosphamide. Crescent J Med Biol Sci.

[B33] Schmittgen TD, Livak KJ (2008). Analyzing real-time PCR data by the comparative CT method. Nat protoc.

[B34] Shah NA, Khan MR (2017). Increase of glutathione, testosterone and antioxidant effects of Jurenia dolomiaea on CCl 4 induced testicular toxicity in rat. BMC Complement. Altern Med.

[B35] Shanmugam G, Narasimhan M, Tamowski S, Darley-Usmar V, Rajasekaran NS (2017). Constitutive activation of Nrf2 induces a stable reductive state in the mouse myocardium. Redox Biol.

[B36] Tayefi‐Nasrabadi H, Sadigh‐Eteghad S, Aghdam Z (2012). The effects of the hydroalcohol extract of Rosa canina fruit on experimentally nephrolithiasic Wistar rats. Phytother Res.

[B37] Winther K, Hansen ASV, Campbell-Tofte J (2016). Bioactive ingredients of rose hips (Rosa canina L) with special reference to antioxidative and anti-inflammatory properties: in vitro studies. Botanics.

[B38] Wyrobek AJ, Gordon LA, Burkhart JG, Francis MW, Kapp Jr RW, Letz G, Malling HV, Topham JC, Whorton MD (1983). An evaluation of the mouse sperm morphology test and other sperm tests in nonhuman mammals: A report of the US Environmental Protection Agency Gene-Tox Program. Mutat Res.

[B39] Young I, Trimble E (1991). Measurement of malondialdehyde in plasma by high performance liquid chromatography with fluorimetric detection. Ann clin biochem.

